# Tracing SARS-CoV-2 Evolution in Algeria: Insights from 2020 to 2023

**DOI:** 10.3390/v18020258

**Published:** 2026-02-18

**Authors:** Fatima Ezzohra Ezahedi, Fawzi Derrar, Ágota Ábrahám, Safia Zeghbib

**Affiliations:** 1 National Laboratory of Virology, Szentágothai Research Centre, University of Pécs, 7622 Pécs, Hungary; fatima.ezahedi@pte.hu (F.E.E.); abraham.agota@pte.hu (Á.Á.); 2 Institute of Biology, Faculty of Sciences, University of Pécs, 7622 Pécs, Hungary; 3 Pasteur Institute of Algeria, Algiers 16047, Algeria; f.derrar@pasteur.dz

**Keywords:** SARS-CoV-2, Algeria, viral mutations

## Abstract

Genomic surveillance is a cornerstone of pandemic response; it has helped guide public health interventions worldwide. However, North Africa stands between limited surveillance resources and efforts to address the data gap in this strategic geographic region that links sub-Saharan Africa and Europe. In this study, we present the first comprehensive evolutionary investigation of Algerian SARS-CoV-2 genomes, revealing their phylogeny, continuous phylogeography within the country, mutation analysis, and a super-spreading event through haplotype network analysis. We characterized the genetic diversity and unique mutation pattern of 449 Algerian sequences, revealing multiple independent introductions into the country since the first reported case on the 25th of February 2020 followed by numerous local transmissions that facilitated the virus’s rapid propagation. This study highlights both the importance of molecular epidemiology and equitable access to resources in implementing genomic epidemiology and in increasing sequencing efforts to strengthen pandemic preparedness.

## 1. Introduction

Despite public health measures to effectively manage the SARS-CoV-2 pandemic and the success of vaccination in easing restrictions, the persistent threat posed by emerging variants remains a significant challenge. Since the emergence of SARS-CoV-2 in 2019, it has become one of the most extensively sequenced pathogens. However, efforts in genomic surveillance have been unevenly distributed across regions worldwide, resulting in geographic gaps.

North Africa, being situated at the intersection of Europe, Africa, and Asia, occupies a strategic position, alongside frequent population movements and trade exchanges. Despite this, genomic and evolutionary surveillance are scarce in this region due to unequal resources and limited awareness. Furthermore, the vaccination rate in Algeria is low: only 17.88% of the population has received at least one dose, reflecting a lack of understanding of safety culture [1]. This limited compliance to pharmaceutical and non-pharmaceutical interventions, including mask use and social distancing, may have contributed to continuous local viral evolution.

Algeria reported its first confirmed SARS-CoV-2 case on 25 February 2020. During the pandemic, genomic sequences were deposited in the GISAID database. A constant delay between sample collection and submission dates was present across the submitted sequences. Nevertheless, the number of high-quality complete genomes suitable for downstream analysis is roughly 16% of the fully available data. On 5 August 2024, Algeria ceased SARS-CoV-2 data sharing. In contrast, other countries continue to submit sequences in full genome length. For instance, Egypt’s most recent submission occurred on 10 December 2025 of a whole-genome sequence collected on 29 October 2025, while Tunisia submitted its latest sequence on 10 October 2025, and Morocco on 11 July 2025 [2].

Previous studies analyzing 29 sequences from Algeria revealed multiple virus introductions despite lockdown. However, the limited sample size has constrained the ability to draw findings on viral evolution and spread within the country [3]. To address these limitations and provide a more comprehensive view of the pandemic in Algeria, 449 complete sequences were obtained from the GISAID database from 2020 to the end of 2023 [2]. Sequences were retrieved using quality criteria appropriate to each analytical framework. Using a combination of phylogenetic and phylogeographic time-based analysis, in addition to local mutation detection, this study aims to evaluate the evolutionary dynamics of SARS-CoV-2 in Algeria to rate the contribution of genomic surveillance as a key component of pandemic preparedness.

## 2. Materials and Methods

### 2.1. Dataset Partitioning

Three datasets were created for different steps of the analysis, the characteristics of each dataset are summarized in Table 1.

### 2.2. Temporal Signal Assessment, Time-Calibrated Phylogeny (Temporal Scaling Phylogeny), and Continuous Phylogeographic Analysis

For this study, 334 Algerian sequences were retrieved from the GISAID database, with collection dates ranging from March 2020 to December 2022. Sequence alignment was performed using the MAFFT web server with fast and rough FFT-NS-2 parameters [4]. Then, a maximum-likelihood tree was generated using IQ-TREE, with the best substitution model selected as GTR + F + R2 [5]. The tree file was used as an input for TempEst to test the temporal signal. Regression analysis of root-to-tip genetic distances with respect to sampling times showed a strong positive correlation (r = 0.96, R^2^ = 0.92), indicating the suitability of the dataset for phylogenetic molecular clock analysis [6]. Subsequently, BEAST v1.10.4 was used to perform a Bayesian phylogenetic analysis and to estimate the evolutionary rate using time-stamped sequences. A relaxed uncorrelated lognormal molecular clock was applied to account for rate variation among lineages, and the substitution rate was inferred in substitutions per site per year. For the nucleotide substitution model, GTR + F was applied with a gamma distribution. For continuous phylogeography, the latitude and longitude of Algerian cities were combined with the Brownian random walk model [7]. TRACER v1.6.0 was used to examine the effective sample size (ESS) [8]. TreeAnnotator v1.10.4 was then applied to annotate the maximum clade credibility (MCC) trees, which were visualized using FigTree v1.4.4 [7,9]. Finally, SpreaD3 v0.9.7 was used to visualize phylogeographic data [10]. To visualize the population density data wrapper was used (https://www.datawrapper.de/).

### 2.3. Genomic Investigation

#### 2.3.1. Mutation Detection

As of January 2024, additional sequences have been added to the GISAID database. A dataset comprising 449 complete Algerian genomes was analyzed using Nextclade (https://clades.nextstrain.org/) to identify mutations specific to viral strains circulating in Algeria and to examine the clustering pattern of Algerian sequences. Furthermore, a sub-dataset containing 193 Algerian sequences, along with the Wuhan reference sequence, EPI_ISL_402124, was created by applying two filters: selecting complete genomes with more than 29,000 nucleotides and manually selecting sequences with less than 2% N chains.

Coding sequences corresponding to ORF1a, ORF1b, S, ORF3a, E, M, ORF6, ORF7a, ORF7b, ORF8, N, and ORF10 were extracted from the subset of 193 Algerian sequences and aligned at the codon level with the goal of assessing the selective pressure. SNAP v2.1.1 was used to perform a pairwise comparison of the codon-aligned nucleotide sequences against the Wuhan reference sequence to determine synonymous (dS) and non-synonymous (dN) substitution rates. Based on this, each gene’s ω ratio (dN/dS) was calculated, with ω > 1 indicating positive selection, ω ≈ 1 neutral evolution, and ω < 1 purifying selection [11,12].

#### 2.3.2. Effects of Mutations on SARS-CoV-2 Proteins

The 449 Algerian sequences were individually analyzed using the CoV-server app in the GISAID database to identify mutational patterns and amino acid variations. PredictSNP was subsequently used to assess the functional impact of these changes on the corresponding proteins [13].

#### 2.3.3. Recombination Screening and Detection

Recombination events were evaluated using the recombination detection program (RDP5). This program analyzes nucleotide sequences by comparing their similarities to detect mosaic sequences that show statistically significant differences [14].

### 2.4. Haplotype Network Construction

The haplotype file in Nexus format was generated using DnaSP v6.12.03 [15]. POPART was then employed to create a graphical representation of the evolutionary relationships among haplotypes, resulting in a haplotype network diagram. The analysis was conducted using 193 complete genomes (no missing data) under the default settings (epsilon = 0) [16].

## 3. Results

### 3.1. Phylogenetic Analysis

**Figure 1:**
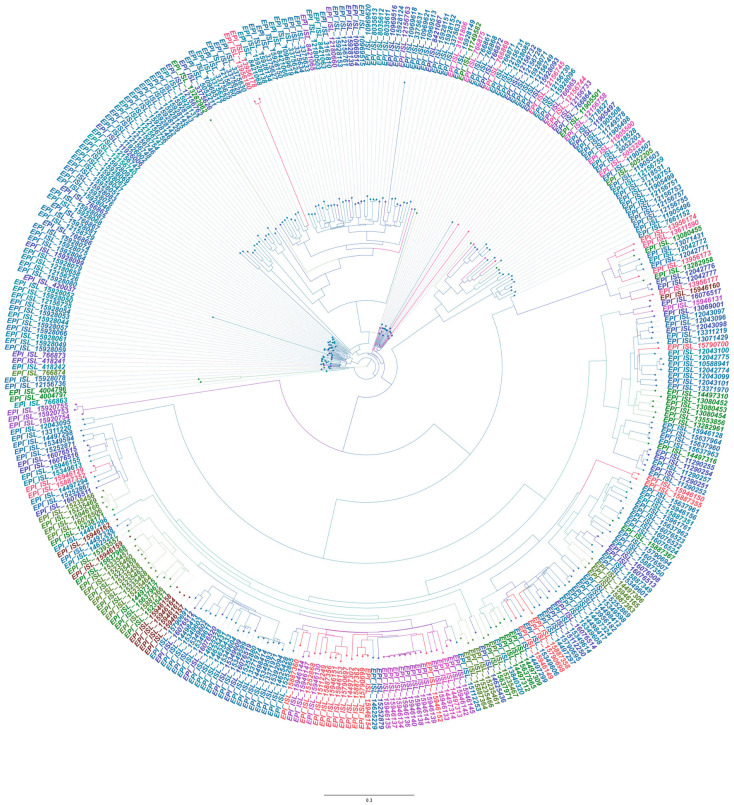
Bayesian phylogenetic tree based on a time-reversible model with unequal rates and unequal empirical base frequencies nucleotide substitution model (GTR + F), using BEAST. Colors indicate posterior, and the values are shown at the end of each node: blue, high posterior probabilities; red, low posterior probabilities. Yellow indicates intermediate values. The tree tips are colored according to their sampling locations.

The estimated evolutionary rate of SARS-CoV-2 in Algeria was 1.43 × 10^−3^ [7.0516 × 10^−4^–1.454 × 10^−3^]. This aligns with the virus’s global estimated evolutionary rate at about 1.1 × 10^−3^ [17]. The phylogenetic analysis revealed substantial diversity, with the sequences forming distinct clusters regardless of their geographic origin, highlighting multiple introductions as shown in Figure 1. Additionally, sequences from the same city were occasionally grouped, emphasizing local circulation and community-level transmission. The incorporation of sampling dates into the analysis enabled the detection of viral trajectories over time, facilitating the tracking of viral spread.

### 3.2. Diversity Among Algerian Sequences

Similarly, based on the Nextclade results displayed in Supplementary Figure S1, both multiple introductions and local transmission were observed. Sequences fell into different clades despite sharing a similar origin; however, in some cases, local clusters were identified in the phylogenetic tree, showing intercity circulation. Furthermore, two sequences (highlighted in grey) were classified as part of a recombinant lineage.

To show the diversity of SARS-CoV-2 lineages circulating locally, all Algerian sequences (1218) available in the GISAID database were examined and subsequently compared with the curated dataset of 449 high-quality genomes. In GISAID, we examined the whole database of available Algerian sequences regardless of quality or length, a total of 140 lineages were identified, with BA.5.2 being the most prevalent (17.75%), followed by B.1.617.2 (13.57%) and BA.2 (10.44%), while 9.63% of sequences remained unassigned. Subsequently, we investigated our dataset composed of 449 sequences; the overall lineage composition was conserved; however, relative frequencies differed, with B.1.617.2 emerging as the dominant lineage (14.89%), followed closely by BA.5.2 (14.61%).To allow a fair comparison between datasets of different sizes and for visualization purposes, only lineages exceeding a proportional frequency threshold of 1.2% were displayed. In contrast, low-frequency lineages were omitted to improve the clarity of the figures (Supplementary Figure S2).

### 3.3. Phylogeographic Diffusion in Continuous Space

**Figure 2:**
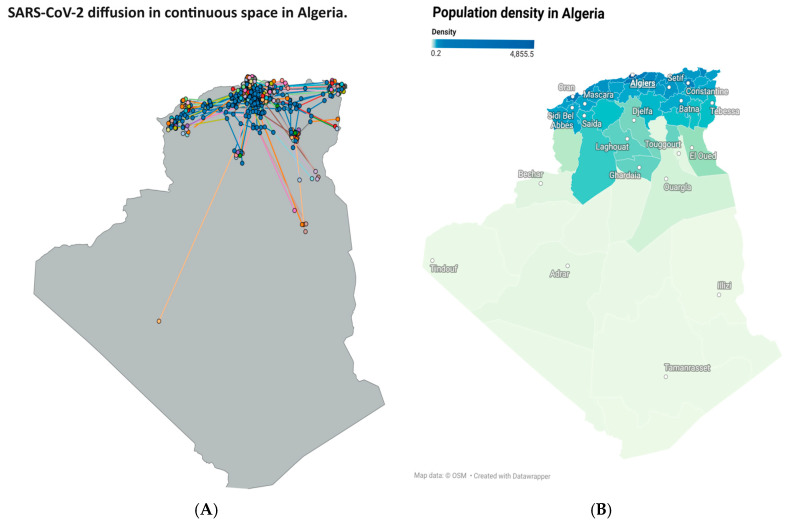
SARS-CoV-2 diffusion in continuous space in relation to population density in Algeria. (**A**) SARS-CoV-2 inferred diffusion routes in continuous geographic space, with blue dots representing internal nodes of the phylogenetic reconstruction, colored lines representing viral lineage movements, and colored dots indicating sampling locations. (**B**) Population density in Algeria, showing the difference between the densely populated northern coastal regions and the sparsely populated desert regions of southern Algeria.

To explore the spread of SARS-CoV-2, a continuous phylogeographic analysis was conducted. This approach is beneficial for reconstructing transmission routes and identifying origins by estimating ancestral viral locations based on the latitudes and longitudes of the sampling sites, together with sampling dates as essential metadata. As a result, the differently colored nodes represent the sampled cities, and the blue nodes indicate internal nodes corresponding to ancestral locations (Figure 2). Alongside, a density map was added Figure 2B to highlight the population differences in different regions.

### 3.4. Phylodynamic Analysis

**Figure 3:**
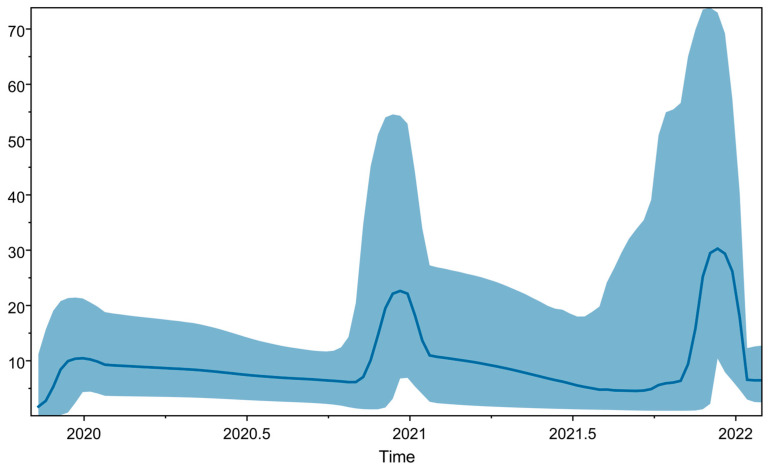
Bayesian skyline plot. Bayesian skyline plot of SARS-CoV-2 Algerian sequences illustrating changes in the adequate population size over time.

The Bayesian skyline plot in Figure 3 provides insights into the population dynamics of SARS-CoV-2 over time, as inferred from the phylogenetic tree. The curve illustrates the changes in adequate population size (Ne) over time. Peaks represent periods of population growth, valleys indicate periods of population decline, and the width of the curve reflects the uncertainty of the Ne estimates. As shown, there are three distinct peaks: the first in 2020, the second in 2021, and the last in 2022, corresponding to the different waves of the pandemic.

### 3.5. Mutation Profile and Selective Pressure Analysis

#### 3.5.1. Selection Pressure

The evolutionary selection pressure for each gene was evaluated using codon-by-codon cumulative behavior plots illustrating synonymous and nonsynonymous substitutions (Supplementary Figure S3) and the calculated values of the nonsynonymous (dN) to synonymous (dS) mutation ratio (ω), summarized in Table 2. The ω ratio (dN/dS) indicates positive selection if it is greater than 1 and adverse selection if it is less than 1. Due to the sensitivity of the selection pressure analysis, a high-quality dataset of 193 sequences was used, which resulted in an imbalance because the number of sequences per SARS-CoV-2 strain was unequal.

Nevertheless, insights into the genes were drawn. As shown in Table 2, ORF1a exhibited a highly adverse selection with a value of 0.26, along with ORF3a, the E gene, ORF6, ORF7a, and ORF8. In contrast, ORF1b, the M gene, and the N gene displayed moderate adverse selection. However, the Spike gene showed a value of 5.06, indicating strong positive selection. For ORF10, no significant results were observed.

The results from SNAP also included plots representing the insertion and deletion ratio (in/del), synonymous mutation rate (syn), and non-synonymous mutation rate (non-syn). Plots were generated for each gene.

As shown in Table 2, ORF1a exhibited a lower rate of non-synonymous substitutions compared to synonymous substitutions. In contrast, some other genes, such as ORF1b, E, M, Spike, and ORF8, showed a higher rate of non-synonymous mutations. In comparison, ORF3a, ORF7a, and ORF6 had a higher frequency of synonymous mutations than non-synonymous mutations.

Specifically, in ORF1a, synonymous substitutions predominated over non-synonymous substitutions. In ORF1b, however, non-synonymous mutations were more frequent (Table 2). Interestingly, the Spike, E, M, N, and ORF8 genes all showed higher rates of non-synonymous substitutions compared to several other genes. In ORF3a, ORF6, and ORF7a, the synonymous mutation rate was higher than the non-synonymous mutation rate. For ORF10, no significant results were observed.

#### 3.5.2. Mutation Profile

Using PredictSNP, mutations present in Algerian sequences were analyzed. The webserver tool integrates data from various methods (MAPP, PhD-SNP, PolyPhen-2, PolyPhen-1, SIFT, SNAP) (Supplementary Table S1) and provides percentages indicating whether a mutation is deleterious (red) or neutral (green).

Using CovServer, each sequence was compared with the Wuhan reference complete genome, and a full analysis of predominant and specific Algerian mutations across sequences was conducted to extract the mutations listed in Supplementary Table S1.

For NSP1, the mutation S135R, which has been present since the start of the pandemic, was classified as deleterious. In NSP2, the most common mutation was Q376K, which was neutral and exclusively found in 2022 sequences. In contrast, NSP3 and NSP6 mutations were recent, with each having one deleterious mutation, G489S and R233C, respectively, while the others were neutral. Similarly, mutations in NSP4, NSP5, NSP13, NSP14, NSP15, E, and NSP7b were neutral and occurred in many recent sequences. Spike mutations were neutral in both early and recent sequences. M and NS8 had one deleterious mutation each, I82T and R52I, respectively, along with additional neutral mutations (Supplementary Table S1). Within NSP12, the mutations T267M and N865H were identified as deleterious. Likewise, mutations in the NS3 protein, including T151I, Q57H, L129F, T223I, and G100C, negatively impacted the protein. Notably, mutations in the nucleocapsid N protein, such as S186Y, S194L, A251V, G204R, G204P, R203M, and S202N, exhibit deleterious effects on its structure and function.

Several mutations specific to the Algerian sequences were identified. In NSP2, leucine (L) was substituted with valine (V) at position 270 in the sequence EPI_ISL_18090022. In NSP3, several mutations were identified, including a substitution of glycine (G) to alanine (A) at position 145 in the sequence EPI_ISL_15946147, phenylalanine (F) to tyrosine (Y) at position 210 in the sequence EPI_ISL_15928079, phenylalanine (F) to isoleucine (I) at position 336 in the sequence EPI_ISL_15235461, and alanine (A) to glycine (G) at position 1431 in the sequence EPI_ISL_12156740. In NSP6, leucine (L) was substituted with valine (V) at position 185 in the sequence EPI_ISL_15928087. In NSP14, a substitution of glycine (G) with serine (S) at position 189 was observed in sequence EPI_ISL_3375626. In NSP15, two mutations were detected: a substitution of lysine (K) to isoleucine (I) at position 307 in the sequence EPI_ISL_15928152 and tryptophan (W) to lysine (K) at position 332 in the sequence EPI_ISL_12156748 (Table 3).

#### 3.5.3. Recombination Detection and Analysis

Recombination detection software revealed one recombination event with strong statistical support. The recombinant sequence was detected using seven of the nine methods, with a significant *p*-value. The results are summarized in Table 4. The sequence in question is EPI_ISL_15920753, sampled from a Male on the 25 September 2022 in the city of Sétif and belonging to the BA.2 sub-lineage of the omicron variant. The Major parent, which, by definition, is the genetically closer parent to the recombinant sequence and contributes to a larger portion of the genome, is EPI_ISL_15790700 sampled on the 18 August 2022 in the city of El_Taref and belongs to the omicron variant sub-lineage BA.2.38. The minor parent was EPI_ISL_12156732 sampled on the 1 October 2020 from Blida and belonging to the B.1.1 lineage from the early pandemic.

### 3.6. Haplotype Network Analysis

**Figure 4:**
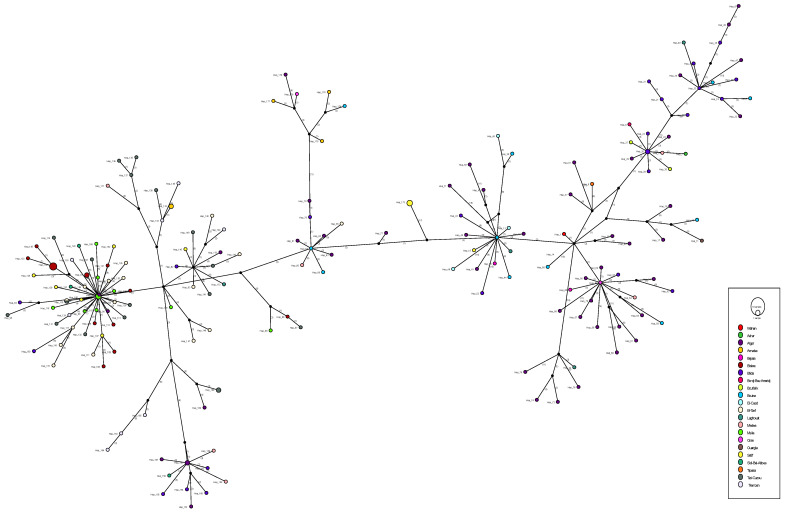
Haplotype network plot of SARS-CoV-2 Algerian sequences. Different colors indicate the sampling locations. The black circles indicate internal nodes pointing to missing unsampled data. The size of the circles is proportional to the number of sequences in each cluster.

Following the genomic investigation, a haplotype analysis was conducted to identify more hidden patterns in the high-quality dataset (193 sequences). The haplotype analysis revealed 179 distinct haplotypes, highlighting the extensive diversity within our dataset (Figure 4). Colored nodes represent the Algerian cities where the samples originated, and black nodes denote internodes, indicating missing or unsampled data. Sequences from the exact location can cluster together, although some diverge significantly despite sharing the same origin. Interestingly, a potential superspreader event can be identified by the central, highly connected nodes observed around H91 in the figure above. The identification of haplotype H91 in distant cities, combined with several directly derived haplotypes collected within a brief time frame, strongly indicates a superspreader event. This could be the result of a highly mobile infected person, combined with a significant gathering that facilitated the virus’s extensive spread.

## 4. Discussion

The emergence of SARS-CoV-2 has highlighted the global importance of genomic sequencing as the central pillar of epidemiological surveillance. Compared to the international data. In Algeria, genomic submissions did not keep pace with the rising number of reported cases, limiting the feasibility of the whole evolutionary pattern. In this study, we demonstrate the importance of sequencing efforts for evolutionary dynamics investigation, molecular tracing, and mutation panel analysis.

### 4.1. Evolutionary Dynamics and Spatiotemporal Spread of SARS-CoV-2 in Algeria

First, when comparing the current mutation rate (1.43 × 10^−3^) to previous estimates, including 18 complete genomes and 11 partial sequences derived from Algeria (5.4043 × 10^−4^), collected at the beginning of the pandemic, a significant increase is clearly observed. This is in line with what is expected for viral fitness improvement and enhancement [18].

Phylogenetic analysis revealed diverse, distinct clades in the dataset, indicating multiple introductions of the virus into Algeria. This was already observed at the beginning of the pandemic when travel restrictions were stringent, and the dataset was smaller [3]. Strikingly, with a larger dataset, the updated phylogeny showed the reclassification of EPI-ISL-766862 with different sequence clustering and tree branching. This sequence from Bouira was previously clustered with EPI_ISL_766866 from the exact location (posterior probability = 80%) [3]; however, in the new analysis, it branched with EPI_ISL_15928087 from Algiers (posterior probability = 100%), which was sampled on the 13 October 2020 and submitted to the GISAID database on the 28th of November 2022. Such differences highlight how additional sampling can refine early assumptions, emphasizing the importance of sampling efforts, sequencing, and real-time reporting in genomic epidemiology. Similarly, sequences from different cities clustered together, demonstrating the impact of local travel on the virus’s spread. While local transmissions at the city level were clearly observed, this indicates the effect of social gatherings on the transmission chain. This occurred alongside the ease or discontinuation of mitigation measures. This is in accordance with the behavioral analysis during the COVID pandemic in Algeria, Tunisia, and Morocco [19].

Similarly, the phylogeographic diffusion analysis in continuous space enabled visualization of the virus’s spread over time and the reconstruction of ancestral locations. Notably, most ancestral locations were in the northern and central parts of Algeria; in the south, only one ancestral location was observed. This pattern correlates with the high population density and travel connectivity in these regions, as well as with sampling bias, where northern and central Algeria have better genomic surveillance than the southern part of the country [20,21]. This geographic bias illustrates that the absence of sequences does not mean the absence of viral circulation, underscoring the need for equitable national sequencing capacity. The Bayesian Skyline demonstrated the ability of sequencing to detect growth dynamics, showing three distinct peaks in population size growth, reflecting different waves driven by the emergence of new variants and increased sequencing efforts [22].

### 4.2. Selection Pressure and Dataset Bias

When evaluating selection pressure across structural, nonstructural, and accessory genes, the S gene showed a significant increase in ω relative to the previous Algerian study [3]. Therefore, this study highlights the impact of larger datasets on increasing statistical power to detect positive or purifying selection. Nonetheless, most sequences were sampled from 2022 and 2023; consequently, the results were influenced by spatiotemporal bias. This manifests as higher ω ratios in the M (0.77) and N (0.86) genes, exceeding typical global estimates (0.1–0.2 for M and 0.2–0.4 for N) [23,24]. This reflects variant-specific adaptation. During 2022–2023, delta and omicron lineages predominated, with mutations such as I82T in M and R203K/G204R in N proteins, which were formerly associated with changes in viral replication, assembly, and immune evasion [25]. Moreover, since the dataset is primarily composed of recent sequences from a limited geographic region, this may influence the reported results. This bias may lead to increased ω values by capturing current founder effects, selective sweeps, or transitory polymorphisms rather than long-term purifying selection pressure [26]. Therefore, it is highly methodologically important to consider temporally and geographically diverse datasets when evaluating selective trends, as these signals are dependent on when and where genomes are sampled.

### 4.3. Functional Mutations and Algeria-Specific Genomic Signature

While examining specific mutations, several deleterious mutations were recognized. The mutation T151I in NS3 (ORF3a) was found to cause structural damage, specifically the breakage of a buried H-bond in both the Alpha and Beta variants, resulting in a loss-of-function mutation [27]. Additionally, the mutation Q57H was found to cause significant structural changes in the dimer conformation [28]. And by extension, other substitutions at structurally relevant residues (such as L129F and G100C) may exert similar functional consequences. Moreover, S194L and S202N identified in N (ORF9) were found to be two effective mutations [29]. Further, the deleterious effects of mutations T267M and N865H, as well as S186Y, A251V, G204R, and S202N in NSP12 and N, respectively, were revealed. One necessary mutation found was R233C in NSP6, which was demonstrated to be deleterious. It was also identified as a missense mutation that alters the function of the NSP6 protein [30]. Additionally, the mutation T223I, found only in recent Algerian sequences and having a deleterious effect, was associated with less efficient replication and may contribute to reduced pathogenicity in the Omicron strain, suggesting that local viral lineages may be attenuated, distinct from global variants [31].

Notably, the analysis of protein sequence mutations revealed nine mutations unique to the Algerian SARS-CoV-2 dataset. Among these, three mutations were detected to have deleterious effects: F336I in NSP3, and K307I and W332K in NSP15. Each of these mutations was identified in a single sequence, EPI_ISL_15235461, EPI_ISL_15928152, and EPI_ISL_12156748, respectively. It was revealed that NSP15 plays a crucial role in host RNA processing, and mutations within this protein may alter viral replication dynamics, potentially affecting viral fitness [32]. Also, studies show that a mutation in NSP3 (S676T) can reduce viral replication by disrupting its interaction with the host’s translation factors. However, a compensatory mutation in the Nucleocapsid protein (N-S194L) can restore virulence [33]. This emphasizes the functional role of NSP3 and its interaction with other proteins in modulating SARS-CoV-2 replication. Remarkably, these mutations have not been previously reported in scientific literature, underscoring the need for further investigation into their functional implications. Hence, we evaluated these mutations using SARS-CoV-2 sequence analysis, within the Coronavirus Antiviral & Resistance Database (https://covdb.stanford.edu/sierra/sars2/by-sequences/), accessed on 15^th^ December 2025, and found no evidence that these unique mutations affect epitope recognition or antiviral susceptibility [34]. Some of these mutations appeared later in sequences worldwide, further reinforcing the need for this initial identification to capture the viral mutation pattern.

### 4.4. Recombinant Events and Dynamic Mutational Pattern

Notably, the Nextclade analysis identified two recombinant sequences in the dataset. The EPI_ISL_12043095 sample, collected in February 2022 from a 76-year-old male, was initially classified by the Pangolin software as the BA.2 omicron subclade. However, during this new investigation, it was found to belong to the XAP recombinant clade. This is a key observation as it predates the official designation of XAP by two months, suggesting earlier cryptic circulation [35]. Similarly, the sequence EPI_ISL_17985292 was collected in April 2023 and belongs to the XBV recombinant clade, which was first reported in March 2023 according to the Outbreak.info reporting system. Nonetheless, according to the GISAID database, it was already circulating in the USA on the 14th of November, 2022 [2,36]. Furthermore, the RDP 5 software detected a third recombinant sequence, EPI_ISL_15920753, from 2022 involving a major parent form sublineage, BA.2.38, and a minor parent from the B.1.1 sublineage. This might be explained by several hypotheses, such as undetected persistence, in which the B.1.1 lineage or its close relative remained present in under-sampled populations or among immunocompromised individuals, making it less likely to be detected in genomic surveillance at the time of the recombination (2022). Similarly, the ancestor proxy effect, where the B.1.1 is not the actual minor parent. Instead, it is the closest known lineage to the actual unsampled parental sequence [37,38]. Such findings illustrate that these lineages can remain concealed in a surveillance-limited setting.

Note that the evolution of SARS-CoV-2 is very dynamic; for instance, mutations that were identified as Algerian characteristic signatures are no longer classified as such due to the growing number of samples and data. Back in 2020, the amino acid replacement A130V in the NSP12 gene was first classified as an Algerian characteristic mutation; however, in recent analyses, it was demonstrated to originate in Abu Dhabi (EPI_ISL_69815) on 12 June 2020. Thereafter, it was imported to Algeria (EPI_ISL_766874) on 21 June 2020. On the other hand, new Algerian substitutions arose, namely the W332K mutation, which was first reported in Algeria on 14 March 2021. This demonstrates the importance of molecular investigation to track new mutations that may affect drug and vaccine development and to develop mitigation measures. Moreover, it enables molecular tracing. For instance, the aforementioned mutation was later spotted in the UK on 26 June 2022. Similarly, the N874H mutation in NSP12, found in EPI-ISL-766875, was determined to be deleterious. In the latest analysis, it was found to have migrated first to the UK on 6 July 2020; however, we demonstrate that its first appearance was actually in Libya on 18 June 2020, then in Egypt on 1 July 2020, and later in the UK. Despite physical barriers and lockdown measures, the virus’s spillover into neighboring countries is evident [2].

### 4.5. Haplotype Network and Evidence of a Superspreading Event

Analysis of the haplotype network revealed high genetic diversity; 197 haplotypes were described within a single country. Two types of clusters were obtained during the haplotype network analysis: those from the exact provenance and others from completely different sampling locations. Specifically, a remarkable cluster centered on a haplotype associated with Algiers and M’sila. It displayed a star-like structure, in which various sequences radiate from a single central node with a small number of mutations. This topology is characteristic of a superspreader event, in which a single infected individual or a small number of individuals engender a large number of secondary cases within a brief timeframe [39,40]. Such events can increase transmission, reduce short-term genetic diversity, and speed up the spread of specific viral variants [41]. These dynamics are evident in the low genetic divergence and restricted mutational branching within the cluster, consistent with rapid dissemination from a single founder. On the other hand, the existence of shared haplotypes across multiple cities suggests internal movement and transmission.

## 5. Conclusions

Overall, our in-depth analysis focused on the evolutionary dynamics of the Algerian SARS-CoV-2 pandemic. Hence, it helps to fill a critical knowledge gap. It emphasizes the importance of real-time genomic surveillance and reporting to support decision making, evaluate diagnostic tools, and guide therapeutic and vaccination strategies. Additionally, it underscores the significant impact of dataset size on the results and conclusions drawn.

This study demonstrates how localized genomic surveillance can generate insights of global relevance. It also highlights that the trajectory of SARS-CoV-2 in Algeria cannot be fully understood without continuous, robust genomic monitoring. Even though moderate-scale sequencing provides actionable data, the limited sample size constrained evolutionary interpretation and underscored broader global inequities in sequencing. Strengthening sequencing networks and implementing strategies for these types of regions might be necessary for future pandemic preparedness.

## Figures and Tables

**Table 1:** Partitioning of Algerian SARS-CoV-2 genomes for downstream analyses.

Number of Sequences	Selection Criteria	Purpose of Analysis
334	Genomes available at the time of temporal signal assessment (passed Quality Control)	Phylogeny and phylogeography
193	High-quality complete genomes (No N chain)	Haplotype network analysis and selection pressure
449	Expanded dataset including later uploads (Only high-quality complete sequences)	Mutation analysis

**Table 2:** Selection pressure of SARS-CoV-2 genes. A summary of the data generated by SNAP, assessing the selective pressure of each gene. ds: observed synonymous substitutions; dn: observed nonsynonymous substitutions. dn/ds: selection pressure.

Gene	ds	dn	dn/ds
ORF1a	0.0023	0.0006	0.26
ORF1b	0.0013	0.0007	0.53
SPIKE	0.0016	0.0081	5.06
ORF3a	0.0056	0.0018	0.32
*E*	0.0179	0.0059	0.32
*M*	0.0068	0.0053	0.77
ORF6	0.0284	0.0067	0.23
ORF7a	0.0120	0.0036	0.3
Orf8	0.0131	0.0035	0.26
*N*	0.0058	0.0050	0.86

**Table 3:** Mutations exclusive to Algerian SARS-CoV-2 sequences. A summary of the Predict SNP results of the classification of Algerian SARS-CoV-2 specific mutations. Red indicates deleterious mutations, while green indicates neutral mutations.

Protein	Mutations	PredictSNP	MAPP	Phd-SNP	PolyPhen-1	PolyPhen-2	SIFT	SNAP
NSP2	L270V	63%	84%	83%	67%	63%	76%	58%
NSP3	G145A	63%	65%	72%	67%	68%	79%	50%
NSP3	F210Y	83%	77%	68%	67%	63%	71%	50%
NSP3	F336I	76%	65%	73%	59%	81%	79%	81%
NSP3	A1431G	83%	73%	83%	67%	61%	61%	50%
NSP6	L185V	75%	64%	78%	67%	63%	76%	71%
NSP14	G189S	74%	75%	72%	67%	41%	76%	67%
NSP15	K307I	87%	86%	73%	74%	54%	79%	81%
NSP15	W332K	87%	92%	88%	74%	81%	79%	89%

**Table 4:** Recombination analysis of 193 high-quality complete Algerian genomes using RDP, GENECONV, Bootscan, Maxchi, Chimaera, SiSscan, and 3Seq methods implemented in the RDP5 software (2021 version).

Breakpoint Position		Recombinant Sequence	Minor Parental Sequence	Major Parental Sequence	Detection Method with a Significant *p* Value						
Begin	End				RDP	GENECONV	Bootscan	Maxchi	Chimaera	SiSscan	3Seq
18,783	26,144	EPI_ISL_15920753	EPI_ISL_12156732	EPI_ISL_15790700	1.42 × 10^−5^	3.48 × 10^−4^	3.43 × 10^−2^	4.11 × 10^−5^	1.03 × 10^−4^	4.04 × 10^−7^	1.5× 10^−4^

## Data Availability

The original contributions presented in this study are included in the article/Supplementary Material. Further inquiries can be directed to the corresponding author.

## Supplementary Materials

The following supporting information can be downloaded at: https://www.mdpi.com/article/10.3390/v18020258/s1, Figure S1: Classification of Algerian sequences in the Nextclade phylo-genetic tree. The phylogeny was generated using Nextclade with GISAID as the database. A total of 449 Algerian sequences were introduced, and the colored dots indicate the clades to which each sequence belongs. The colors specified in the legend represent different clades, with grey indicating a recombinant sequence. Figure S2: Lineage distribution of Algerian SARS-CoV-2 sequences. (A) Lineage composition of all 1218 Algerian SARS-CoV-2 sequences available in the GISAID database at the time of analysis. (B) Lineage composition of the curated dataset of 449 high-quality genomes. To enable meaningful comparisons between datasets of different sizes, lineage-frequency thresholds were scaled proportionally, and lineages representing fewer than approximately 1.2% of sequences were omitted. Each color represents a distinct lineage, or-dered in the legend by decreasing relative frequency. Figure S3: Plots of insertion/deletion, synonymous, and nonsynonymous mutation rates for each nucleotide in the genes. The plot illustrates how genetic changes (substitutions) occur in two specific types, synonymous and nonsynonymous, across each analyzed gene. The accumulation of amino acid substitutions in 193 Algerian sequences was examined, including the SARS-CoV-2 reference sequence from Wuhan, China. In the graph, the X-axis represents the codons, and the Y-axis represents the number of substitutions. The red line corresponds to synonymous substitutions, the green line represents nonsynonymous substitutions, and the gray vertical line marks stop codons. Table S1: Mutations in SARS-CoV-2 proteins (complete set of Algerian sequences). Red indicates deleterious mutations, while green indicates neutral mutations.

## Abbreviations

The following abbreviations are used in this manuscript:

BEAST	Bayesian Evolutionary Analysis Sampling Trees
dN	Nonsynonymous substitution rate
dS	Synonymous substitution rate
ESS	Effective Sample Size
FFT-NS-2	Fast Fourier-Transform-based alignment algorithm
GISAID	Global Initiative on Sharing All Influenza Data
GTR	General Time Reversible model
IQ-TREE	Efficient Phylogenetic Tree Reconstruction software
MAFFT	Multiple Alignment using Fast Fourier Transform
MCC	Maximum Clade Credibility
Ne	Effective population size
NSP	Nonstructural protein
ORF	Open reading frame
RDP	Recombination Detection Program
SARS-CoV-2	Severe Acute Respiratory Syndrome Coronavirus 2
SNAP	Synonymous Non-synonymous Analysis Program
