# Supplementary material for: Tracing SARS-CoV-2 Evolution in Algeria: Insights from 2020 to 2023
Source: Viruses. 2026 Feb 18;18(2):258. doi: 10.3390/v18020258 (PMC12945117; doi:10.3390/v18020258)
Supplement: Supplementary file 1 [file viruses-18-00258-s001.zip › Id and dates 449.pdf]

Accession ID	Collection date	Submission date	Location
EPI_ISL_10588941	1/31/2022	3/2/2022	Algiers
EPI_ISL_10969513	12/14/2021	3/13/2022	Blida
EPI_ISL_10969514	12/14/2021	3/13/2022	Bouira
EPI_ISL_10969515	12/15/2021	3/13/2022	Algiers
EPI_ISL_10969516	12/15/2021	3/13/2022	Bouira
EPI_ISL_10969517	12/21/2021	3/13/2022	Algiers
EPI_ISL_10969618	1/2/2022	3/13/2022	Algiers
EPI_ISL_10969619	12/29/2021	3/13/2022	Algiers
EPI_ISL_10969620	12/29/2021	3/13/2022	Algiers
EPI_ISL_10969621	12/29/2021	3/13/2022	Algiers
EPI_ISL_11290251	2/26/2022	3/22/2022	Bouira
EPI_ISL_11290252	2/27/2022	3/22/2022	Algiers
EPI_ISL_11290254	2/27/2022	3/22/2022	Bouira
EPI_ISL_11290255	2/27/2022	3/22/2022	Bouira
EPI_ISL_11290257	2/28/2022	3/22/2022	Algiers
EPI_ISL_11749578	5/25/2021	8/26/2021	Algiers
EPI_ISL_11749582	5/3/2021	8/26/2021	Oran
EPI_ISL_11905496	2/17/2021	4/10/2022	Algiers
EPI_ISL_11905497	5/2/2021	4/10/2022	Bouira
EPI_ISL_11905498	5/27/2021	4/10/2022	Algiers
EPI_ISL_11905499	5/31/2021	4/10/2022	Algiers
EPI_ISL_11905500	5/1/2021	4/10/2022	Ouargla
EPI_ISL_11905501	5/3/2021	4/10/2022	Oran
EPI_ISL_11905503	5/10/2021	4/10/2022	Algiers
EPI_ISL_11905507	5/31/2021	4/10/2022	Algiers
EPI_ISL_11905508	5/31/2021	4/10/2022	Algiers
EPI_ISL_11926002	5/24/2021	4/10/2022	Algiers
EPI_ISL_11926006	4/27/2021	4/10/2022	Bouira
EPI_ISL_12042771	2/21/2022	4/14/2022	Algiers
EPI_ISL_12042772	2/21/2022	4/14/2022	Medea
EPI_ISL_12042774	2/24/2022	4/14/2022	Algiers
EPI_ISL_12042775	2/24/2022	4/14/2022	Algiers
EPI_ISL_12042776	2/27/2022	4/14/2022	Bouira
EPI_ISL_12042777	2/27/2022	4/14/2022	Bouira
EPI_ISL_12043095	2/6/2022	4/14/2022	Blida
EPI_ISL_12043096	2/6/2022	4/14/2022	Algiers
EPI_ISL_12043097	2/6/2022	4/14/2022	Algiers
EPI_ISL_12043098	2/6/2022	4/14/2022	Bouira
EPI_ISL_12043099	2/7/2022	4/14/2022	Algiers
EPI_ISL_12043100	2/7/2022	4/14/2022	Medea
EPI_ISL_12043101	2/10/2022	4/14/2022	Algiers
EPI_ISL_12156725	5/2/2020	4/20/2022	Medea
EPI_ISL_12156726	5/16/2020	4/20/2022	Blida

EPI_ISL_12156728	9/19/2020	4/20/2022	Algiers
EPI_ISL_12156729	9/20/2020	4/20/2022	Algiers
EPI_ISL_12156730	9/21/2020	4/20/2022	Algiers
EPI_ISL_12156731	9/29/2020	4/20/2022	Blida
EPI_ISL_12156732	10/1/2020	4/20/2022	Bouira
EPI_ISL_12156733	10/3/2020	4/20/2022	Bejaia
EPI_ISL_12156736	11/29/2020	4/20/2022	Algiers
EPI_ISL_12156740	12/24/2020	4/20/2022	Tipaza
EPI_ISL_12156742	12/29/2020	4/20/2022	Blida
EPI_ISL_12156744	1/25/2021	4/20/2022	El Oued
EPI_ISL_12156745	1/25/2021	4/20/2022	El Oued
EPI_ISL_12156746	3/1/2021	4/20/2022	Algiers
EPI_ISL_12156747	3/13/2021	4/20/2022	Laghouat
EPI_ISL_12156748	3/14/2021	4/20/2022	Algiers
EPI_ISL_12156749	3/22/2021	4/20/2022	Blida
EPI_ISL_12156751	4/26/2021	4/20/2022	Algiers
EPI_ISL_12156752	4/26/2021	4/20/2022	Algiers
EPI_ISL_12156753	4/28/2021	4/20/2022	Algiers
EPI_ISL_12156754	5/2/2021	4/20/2022	Algiers
EPI_ISL_12156755	5/9/2021	4/20/2022	Algiers
EPI_ISL_12156756	5/11/2021	4/20/2022	Algiers
EPI_ISL_12156757	6/13/2021	4/20/2022	Algiers
EPI_ISL_12156758	7/6/2021	4/20/2022	Hassi Messaoud
EPI_ISL_12156759	7/6/2021	4/20/2022	Laghouat
EPI_ISL_12156760	7/7/2021	4/20/2022	Blida
EPI_ISL_12156761	9/7/2021	4/20/2022	Algiers
EPI_ISL_12156762	9/13/2021	4/20/2022	Bouira
EPI_ISL_12156763	9/16/2021	4/20/2022	Bejaia
EPI_ISL_12180503	12/28/2021	3/13/2022	Blida
EPI_ISL_12180658	1/2/2021	4/20/2022	Laghouat
EPI_ISL_12180660	9/18/2021	4/20/2022	Bejaia
EPI_ISL_13069001	3/17/2022	6/2/2022	Bouira
EPI_ISL_13071429	4/18/2022	6/2/2022	Algiers
EPI_ISL_13071431	4/20/2022	6/2/2022	Algiers
EPI_ISL_13080452	2/28/2022	6/2/2022	Oran
EPI_ISL_13080453	3/7/2022	6/2/2022	Oran
EPI_ISL_13080454	3/8/2022	6/2/2022	Oran
EPI_ISL_13080455	3/10/2022	6/2/2022	Oran
EPI_ISL_13282958	1/11/2022	6/14/2022	Oran
EPI_ISL_13282961	2/15/2022	6/14/2022	Oran
EPI_ISL_13282962	2/16/2022	6/14/2022	Oran
EPI_ISL_13311219	6/1/2022	6/15/2022	Algiers
EPI_ISL_13311220	6/4/2022	6/15/2022	Algiers
EPI_ISL_13371970	6/12/2022	6/20/2022	Algiers

EPI_ISL_13371971	6/12/2022	6/20/2022	Algiers
EPI_ISL_13371974	6/12/2022	6/20/2022	Algiers
EPI_ISL_13553856	5/18/2022	6/30/2022	Aidi Bel Abbes
EPI_ISL_13611590	2/15/2022	7/3/2022	Annaba
EPI_ISL_13840320	7/8/2022	7/15/2022	Laghouat
EPI_ISL_13956173	2/2/2022	7/20/2022	Annaba
EPI_ISL_13956174	1/23/2022	7/20/2022	Annaba
EPI_ISL_13956177	1/26/2022	7/20/2022	Annaba
EPI_ISL_13956178	1/7/2022	7/20/2022	Annaba
EPI_ISL_13956180	1/5/2022	7/20/2022	Annaba
EPI_ISL_13956182	6/27/2022	7/20/2022	Algiers
EPI_ISL_14497294	7/20/2022	8/17/2022	Algiers
EPI_ISL_14497296	7/18/2022	8/17/2022	Blida
EPI_ISL_14497297	7/21/2022	8/17/2022	Algiers
EPI_ISL_14497298	7/18/2022	8/17/2022	Algiers
EPI_ISL_14497299	7/18/2022	8/17/2022	Algiers
EPI_ISL_14497302	7/19/2022	8/17/2022	Tebessa
EPI_ISL_14497306	6/29/2022	8/17/2022	Tlemcen
EPI_ISL_14497309	7/4/2022	8/17/2022	Oran
EPI_ISL_14497310	7/10/2022	8/17/2022	Oran
EPI_ISL_14497312	7/12/2022	8/17/2022	Oran
EPI_ISL_14497313	6/29/2022	8/17/2022	Ouargla
EPI_ISL_14497314	7/17/2022	8/17/2022	Touggourt
EPI_ISL_14497315	6/30/2022	8/17/2022	Oran
EPI_ISL_14497316	7/17/2022	8/17/2022	Oran
EPI_ISL_14497324	7/26/2022	8/17/2022	Algiers
EPI_ISL_14497328	7/27/2022	8/17/2022	Algiers
EPI_ISL_14498412	7/20/2022	8/17/2022	Algiers
EPI_ISL_14498413	7/20/2022	8/17/2022	Algiers
EPI_ISL_14498414	7/20/2022	8/17/2022	Algiers
EPI_ISL_14498417	7/17/2022	8/17/2022	Algiers
EPI_ISL_14625227	8/2/2022	8/25/2022	Blida
EPI_ISL_14625228	8/3/2022	8/25/2022	MEDEA
EPI_ISL_14625229	8/14/2022	8/25/2022	Algiers
EPI_ISL_14625428	8/1/2022	8/25/2022	MEDEA
EPI_ISL_14625431	8/1/2022	8/25/2022	MEDEA
EPI_ISL_14625433	8/4/2022	8/25/2022	MEDEA
EPI_ISL_14625436	8/3/2022	8/25/2022	Bouira
EPI_ISL_15112249	9/7/2022	9/26/2022	Tebessa
EPI_ISL_15112250	9/8/2022	9/26/2022	Bouira
EPI_ISL_15112253	9/11/2022	9/26/2022	Algiers
EPI_ISL_15235456	7/14/2022	10/4/2022	Tlemcen
EPI_ISL_15235457	7/18/2022	10/4/2022	Tlemcen
EPI_ISL_15235458	7/19/2022	10/4/2022	Tlemcen

EPI_ISL_15235459	7/19/2022	10/4/2022	Tlemcen
EPI_ISL_15235460	7/19/2022	10/4/2022	Tlemcen
EPI_ISL_15235461	7/20/2022	10/4/2022	Tlemcen
EPI_ISL_15235463	7/20/2022	10/4/2022	Tlemcen
EPI_ISL_15235464	7/21/2022	10/4/2022	Tlemcen
EPI_ISL_15235466	7/24/2022	10/4/2022	Tlemcen
EPI_ISL_15235467	7/24/2022	10/4/2022	Tlemcen
EPI_ISL_15235468	7/25/2022	10/4/2022	Tlemcen
EPI_ISL_15235469	7/25/2022	10/4/2022	Tlemcen
EPI_ISL_15235471	7/24/2022	10/4/2022	Oran
EPI_ISL_15238171	8/14/2022	10/4/2022	Oran
EPI_ISL_15252865	8/21/2022	10/5/2022	Algiers
EPI_ISL_15252867	8/15/2022	10/5/2022	Algiers
EPI_ISL_15252868	8/15/2022	10/5/2022	Tebessa
EPI_ISL_15252869	8/18/2022	10/5/2022	Algiers
EPI_ISL_15252871	8/17/2022	10/5/2022	Algiers
EPI_ISL_15252879	8/16/2022	10/5/2022	Bouira
EPI_ISL_15252880	8/15/2022	10/5/2022	Algiers
EPI_ISL_15349593	8/14/2022	10/13/2022	Algiers
EPI_ISL_15349594	8/15/2022	10/13/2022	Algiers
EPI_ISL_15349598	8/13/2022	10/13/2022	Algiers
EPI_ISL_15349599	8/13/2022	10/13/2022	Algiers
EPI_ISL_15349601	8/17/2022	10/13/2022	Algiers
EPI_ISL_15349602	8/17/2022	10/13/2022	Algiers
EPI_ISL_15349603	8/17/2022	10/13/2022	Algiers
EPI_ISL_15349604	8/21/2022	10/13/2022	Algiers
EPI_ISL_15349606	8/21/2022	10/13/2022	Algiers
EPI_ISL_15349608	8/22/2022	10/13/2022	Blida
EPI_ISL_15349609	8/16/2022	10/13/2022	Bouira
EPI_ISL_15349611	8/14/2022	10/13/2022	Medea
EPI_ISL_15349613	8/15/2022	10/13/2022	Medea
EPI_ISL_15349614	8/21/2022	10/13/2022	Medea
EPI_ISL_15392094	9/26/2022	10/17/2022	Algiers
EPI_ISL_15393557	8/8/2022	10/17/2022	Oran
EPI_ISL_15637960	10/12/2022	11/3/2022	Algiers
EPI_ISL_15637961	10/5/2022	11/3/2022	Algiers
EPI_ISL_15637962	10/4/2022	11/3/2022	Algiers
EPI_ISL_15637963	10/15/2022	11/3/2022	Algiers
EPI_ISL_15637964	10/15/2022	11/3/2022	Algiers
EPI_ISL_15637965	9/8/2022	11/3/2022	Oran
EPI_ISL_15637967	8/17/2022	11/3/2022	Oran
EPI_ISL_15637968	8/7/2022	11/3/2022	Sidi Bel Abbes
EPI_ISL_15637971	7/27/2022	11/3/2022	Sidi Bel Abbes
EPI_ISL_15790691	10/4/2022	11/16/2022	Blida

EPI_ISL_15790694	10/12/2022	11/16/2022	Blida
EPI_ISL_15790695	10/24/2022	11/16/2022	Algiers
EPI_ISL_15790697	8/10/2022	11/16/2022	El Tarf
EPI_ISL_15790698	8/10/2022	11/16/2022	El Tarf
EPI_ISL_15790699	8/10/2022	11/16/2022	El Tarf
EPI_ISL_15790700	8/18/2022	11/16/2022	El Tarf
EPI_ISL_15887349	11/3/2022	11/23/2022	Algiers
EPI_ISL_15887350	11/7/2022	11/23/2022	Blida
EPI_ISL_15887351	11/7/2022	11/23/2022	Blida
EPI_ISL_15887352	6/26/2022	11/23/2022	Annaba
EPI_ISL_15887355	7/17/2022	11/23/2022	El Tarf
EPI_ISL_15887356	7/20/2022	11/23/2022	El Tarf
EPI_ISL_15887358	7/31/2022	11/23/2022	El Tarf
EPI_ISL_15887360	8/1/2022	11/23/2022	El Tarf
EPI_ISL_15887362	8/8/2022	11/23/2022	El Tarf
EPI_ISL_15887363	7/31/2022	11/23/2022	Tlemcen
EPI_ISL_15887364	8/4/2022	11/23/2022	Tlemcen
EPI_ISL_15887366	8/8/2022	11/23/2022	Tlemcen
EPI_ISL_15887367	10/12/2022	11/23/2022	Sidi Bel Abbes
EPI_ISL_15920753	9/25/2022	11/27/2022	Setif
EPI_ISL_15920754	10/5/2022	11/27/2022	Setif
EPI_ISL_15920755	10/20/2022	11/27/2022	Setif
EPI_ISL_15928044	3/19/2020	11/28/2022	Blida
EPI_ISL_15928046	3/19/2020	11/28/2022	Blida
EPI_ISL_15928047	3/19/2020	11/28/2022	Blida
EPI_ISL_15928049	3/24/2020	11/28/2022	Blida
EPI_ISL_15928050	3/25/2020	11/28/2022	Blida
EPI_ISL_15928051	3/27/2020	11/28/2022	Blida
EPI_ISL_15928052	3/27/2020	11/28/2022	Blida
EPI_ISL_15928053	3/29/2020	11/28/2022	Blida
EPI_ISL_15928054	3/29/2020	11/28/2022	Blida
EPI_ISL_15928057	4/29/2020	11/28/2022	Algiers
EPI_ISL_15928059	4/30/2020	11/28/2022	Algiers
EPI_ISL_15928061	5/2/2020	11/28/2022	Blida
EPI_ISL_15928064	5/8/2020	11/28/2022	Blida
EPI_ISL_15928066	5/15/2020	11/28/2022	Algiers
EPI_ISL_15928067	5/14/2020	11/28/2022	Blida
EPI_ISL_15928068	5/14/2020	11/28/2022	Blida
EPI_ISL_15928069	5/16/2020	11/28/2022	Algiers
EPI_ISL_15928071	6/19/2020	11/28/2022	Algiers
EPI_ISL_15928074	6/30/2020	11/28/2022	Algiers
EPI_ISL_15928075	6/30/2020	11/28/2022	Algiers
EPI_ISL_15928076	6/30/2020	11/28/2022	Algiers
EPI_ISL_15928078	7/5/2020	11/28/2022	Algiers

EPI_ISL_15928079	7/3/2020	11/28/2022	Blida
EPI_ISL_15928080	7/3/2020	11/28/2022	Blida
EPI_ISL_15928081	7/6/2020	11/28/2022	Algiers
EPI_ISL_15928083	8/5/2020	11/28/2022	Algiers
EPI_ISL_15928085	9/20/2020	11/28/2022	Algiers
EPI_ISL_15928087	10/13/2020	11/28/2022	Algiers
EPI_ISL_15928092	11/24/2020	11/28/2022	Algiers
EPI_ISL_15928093	11/29/2020	11/28/2022	Bouira
EPI_ISL_15928096	12/7/2020	11/28/2022	Algiers
EPI_ISL_15928099	12/13/2020	11/28/2022	Bouira
EPI_ISL_15928124	8/26/2021	11/28/2022	Algiers
EPI_ISL_15928125	9/13/2021	11/28/2022	Blida
EPI_ISL_15928129	10/6/2021	11/28/2022	Algiers
EPI_ISL_15928134	11/2/2021	11/28/2022	Algiers
EPI_ISL_15928137	11/3/2021	11/28/2022	Medea
EPI_ISL_15928138	11/4/2021	11/28/2022	Algiers
EPI_ISL_15928139	11/10/2021	11/28/2022	Bouira
EPI_ISL_15928142	11/28/2021	11/28/2022	Algiers
EPI_ISL_15928143	11/28/2021	11/28/2022	Algiers
EPI_ISL_15928145	12/12/2021	11/28/2022	Algiers
EPI_ISL_15928146	12/12/2021	11/28/2022	Algiers
EPI_ISL_15928149	12/12/2021	11/28/2022	Algiers
EPI_ISL_15928150	12/23/2021	11/28/2022	Algiers
EPI_ISL_15928151	12/23/2021	11/28/2022	Algiers
EPI_ISL_15928152	12/23/2021	11/28/2022	Algiers
EPI_ISL_15928153	12/26/2021	11/28/2022	Algiers
EPI_ISL_15946128	11/13/2022	11/29/2022	Algiers
EPI_ISL_15946129	7/12/2022	11/29/2022	Annaba
EPI_ISL_15946130	7/13/2022	11/29/2022	Biskra
EPI_ISL_15946131	7/14/2022	11/29/2022	Biskra
EPI_ISL_15946133	7/17/2022	11/29/2022	Biskra
EPI_ISL_15946134	7/13/2022	11/29/2022	Biskra
EPI_ISL_15946135	7/13/2022	11/29/2022	Biskra
EPI_ISL_15946136	7/13/2022	11/29/2022	Biskra
EPI_ISL_15946137	7/13/2022	11/29/2022	Biskra
EPI_ISL_15946138	7/13/2022	11/29/2022	Biskra
EPI_ISL_15946139	7/13/2022	11/29/2022	Biskra
EPI_ISL_15946140	7/13/2022	11/29/2022	Biskra
EPI_ISL_15946141	7/13/2022	11/29/2022	Biskra
EPI_ISL_15946142	7/13/2022	11/29/2022	Biskra
EPI_ISL_15946144	7/31/2022	11/29/2022	Biskra
EPI_ISL_15946145	8/1/2022	11/29/2022	Biskra
EPI_ISL_15946147	7/26/2022	11/29/2022	Biskra
EPI_ISL_15946149	7/18/2022	11/29/2022	El Taref

EPI_ISL_15946150	7/18/2022	11/29/2022	El Taref
EPI_ISL_15946151	7/19/2022	11/29/2022	El Taref
EPI_ISL_15946152	7/19/2022	11/29/2022	El Taref
EPI_ISL_15946153	7/28/2022	11/29/2022	El Taref
EPI_ISL_15946154	8/8/2022	11/29/2022	El Taref
EPI_ISL_15946155	11/14/2022	11/29/2022	Medea
EPI_ISL_15946156	11/15/2022	11/29/2022	Medea
EPI_ISL_15946157	7/13/2022	11/29/2022	Msila
EPI_ISL_15946158	7/13/2022	11/29/2022	Msila
EPI_ISL_15946159	7/14/2022	11/29/2022	Msila
EPI_ISL_15946160	7/14/2022	11/29/2022	Msila
EPI_ISL_15946161	7/18/2022	11/29/2022	Msila
EPI_ISL_15946162	7/18/2022	11/29/2022	Msila
EPI_ISL_15946164	7/19/2022	11/29/2022	Msila
EPI_ISL_15946165	7/31/2022	11/29/2022	Tlemcen
EPI_ISL_15946166	7/30/2022	11/29/2022	Tlemcen
EPI_ISL_15961761	11/13/2022	11/29/2022	Algiers
EPI_ISL_16076506	7/26/2022	12/12/2022	Tlemcen
EPI_ISL_16076507	11/23/2022	12/12/2022	Algiers
EPI_ISL_16076508	8/2/2022	12/12/2022	Tizi-Ouzou
EPI_ISL_16076511	7/28/2022	12/12/2022	Tizi-Ouzou
EPI_ISL_16076512	7/29/2022	12/12/2022	Tizi-Ouzou
EPI_ISL_16076513	7/29/2022	12/12/2022	Tizi-Ouzou
EPI_ISL_16076514	8/9/2022	12/12/2022	Tizi-Ouzou
EPI_ISL_16076515	8/10/2022	12/12/2022	Tizi-Ouzou
EPI_ISL_16076516	8/10/2022	12/12/2022	Tizi-Ouzou
EPI_ISL_16076517	8/10/2022	12/12/2022	Tizi-Ouzou
EPI_ISL_16076519	8/1/2022	12/12/2022	Tizi-Ouzou
EPI_ISL_16076520	8/1/2022	12/12/2022	Tizi-Ouzou
EPI_ISL_16076522	11/20/2022	12/12/2022	Algiers
EPI_ISL_16076523	11/19/2022	12/12/2022	Algiers
EPI_ISL_16076524	11/23/2022	12/12/2022	Algiers
EPI_ISL_16242279	12/5/2022	12/22/2022	Algiers
EPI_ISL_16242280	12/4/2022	12/22/2022	Algiers
EPI_ISL_16242281	12/3/2022	12/22/2022	Algiers
EPI_ISL_16242282	12/7/2022	12/22/2022	Algiers
EPI_ISL_16242283	12/8/2022	12/22/2022	Algiers
EPI_ISL_16242284	12/7/2022	12/22/2022	Algiers
EPI_ISL_16242287	12/6/2022	12/22/2022	Blida
EPI_ISL_16242288	11/22/2022	12/22/2022	Medea
EPI_ISL_16242289	12/5/2022	12/22/2022	Medea
EPI_ISL_17182680	12/6/2022	3/11/2023	Algiers
EPI_ISL_17182681	12/7/2022	3/11/2023	Algiers
EPI_ISL_17182682	12/22/2022	3/11/2023	Algiers

EPI_ISL_17182683	12/21/2022	3/11/2023	Algiers
EPI_ISL_17182685	12/26/2022	3/11/2023	Blida
EPI_ISL_17182687	12/24/2022	3/11/2023	Algiers
EPI_ISL_17182688	12/30/2022	3/11/2023	Algiers
EPI_ISL_17182689	12/29/2022	3/11/2023	Algiers
EPI_ISL_17182690	12/28/2022	3/11/2023	Algiers
EPI_ISL_17182691	12/28/2022	3/11/2023	Algiers
EPI_ISL_17182692	12/28/2022	3/11/2023	Algiers
EPI_ISL_17182693	12/28/2022	3/11/2023	Algiers
EPI_ISL_17182695	1/3/2023	3/11/2023	Algiers
EPI_ISL_17182696	1/3/2023	3/11/2023	Algiers
EPI_ISL_17182697	1/4/2023	3/11/2023	Algiers
EPI_ISL_17182698	1/8/2023	3/11/2023	Algiers
EPI_ISL_17182700	1/12/2023	3/11/2023	Algiers
EPI_ISL_17182701	1/15/2023	3/11/2023	Blida
EPI_ISL_17182702	1/17/2023	3/11/2023	Medea
EPI_ISL_17182703	1/17/2023	3/11/2023	Algiers
EPI_ISL_17182705	1/23/2023	3/11/2023	Algiers
EPI_ISL_17182706	1/23/2023	3/11/2023	Algiers
EPI_ISL_17182709	2/8/2023	3/11/2023	Algiers
EPI_ISL_17182710	2/19/2023	3/11/2023	Algiers
EPI_ISL_17222029	12/18/2022	3/16/2023	Bejaia
EPI_ISL_17675167	11/24/2022	5/17/2023	Algiers
EPI_ISL_17675168	12/12/2022	5/17/2023	Algiers
EPI_ISL_17675169	12/15/2022	5/17/2023	Algiers
EPI_ISL_17675172	12/13/2022	5/17/2023	Algiers
EPI_ISL_17675173	12/13/2022	5/17/2023	Algiers
EPI_ISL_17985280	5/8/2023	7/14/2023	Medea
EPI_ISL_17985281	4/3/2023	7/14/2023	Oran
EPI_ISL_17985282	4/9/2023	7/14/2023	Oran
EPI_ISL_17985283	4/9/2023	7/14/2023	Oran
EPI_ISL_17985284	4/18/2023	7/14/2023	Oran
EPI_ISL_17985285	4/25/2023	7/14/2023	Oran
EPI_ISL_17985286	4/25/2023	7/14/2023	Oran
EPI_ISL_17985287	4/27/2023	7/14/2023	Oran
EPI_ISL_17985289	4/29/2023	7/14/2023	Oran
EPI_ISL_17985290	4/5/2023	7/14/2023	Oran
EPI_ISL_17985291	4/15/2023	7/14/2023	Oran
EPI_ISL_17985292	4/17/2023	7/14/2023	Oran
EPI_ISL_17985293	4/17/2023	7/14/2023	Oran
EPI_ISL_17985294	4/18/2023	7/14/2023	Oran
EPI_ISL_17985295	4/26/2023	7/14/2023	Oran
EPI_ISL_17985296	5/4/2023	7/14/2023	Oran
EPI_ISL_17986356	4/18/2023	7/14/2023	Blida

EPI_ISL_17986357	4/24/2023	7/14/2023	Tissemsilt
EPI_ISL_17986358	4/30/2023	7/14/2023	Medea
EPI_ISL_17986359	4/29/2023	7/14/2023	Medea
EPI_ISL_17986360	5/3/2023	7/14/2023	Blida
EPI_ISL_17986361	3/20/2023	7/14/2023	Tebessa
EPI_ISL_17986362	3/26/2023	7/14/2023	Algiers
EPI_ISL_17986363	4/1/2023	7/14/2023	Algiers
EPI_ISL_17986364	4/4/2023	7/14/2023	Algiers
EPI_ISL_18090022	4/30/2023	8/10/2023	Medea
EPI_ISL_18090023	4/26/2023	8/10/2023	Tebessa
EPI_ISL_18090024	4/26/2023	8/10/2023	Algiers
EPI_ISL_18090025	3/29/2023	8/10/2023	Algiers
EPI_ISL_18090027	5/19/2023	8/10/2023	Algiers
EPI_ISL_18090028	3/26/2023	8/10/2023	Oran
EPI_ISL_18090029	4/16/2023	8/10/2023	Oran
EPI_ISL_18090030	4/11/2023	8/10/2023	Oran
EPI_ISL_18090031	4/27/2023	8/10/2023	Oran
EPI_ISL_18090032	5/6/2023	8/10/2023	Algiers
EPI_ISL_18350779	7/4/2022	1/9/2023	Biskra
EPI_ISL_18830340	1/9/2023	1/27/2024	Tizi-Ouzou
EPI_ISL_18830341	1/25/2023	1/27/2024	Tizi-Ouzou
EPI_ISL_18830342	1/2/2023	1/27/2024	Tizi-Ouzou
EPI_ISL_18830343	1/19/2023	1/27/2024	Tizi-Ouzou
EPI_ISL_18830344	2/24/2023	1/27/2024	Tizi-Ouzou
EPI_ISL_18830345	2/6/2023	1/27/2024	Tizi-Ouzou
EPI_ISL_18830346	2/13/2023	1/27/2024	Tebessa
EPI_ISL_18830347	2/20/2023	1/27/2024	Algiers
EPI_ISL_18830348	2/27/2023	1/27/2024	Algiers
EPI_ISL_18830349	3/2/2023	1/27/2024	Algiers
EPI_ISL_18830350	3/2/2023	1/27/2024	Tebessa
EPI_ISL_18830351	3/10/2023	1/27/2024	Algiers
EPI_ISL_18830352	3/13/2023	1/27/2024	Algiers
EPI_ISL_18830353	3/2/2023	1/27/2024	Oran
EPI_ISL_18830354	3/2/2023	1/27/2024	Oran
EPI_ISL_18830355	3/2/2023	1/27/2024	Oran
EPI_ISL_18830356	3/2/2023	1/27/2024	Oran
EPI_ISL_18830357	3/15/2023	1/27/2024	Algiers
EPI_ISL_18830358	3/16/2023	1/27/2024	Algiers
EPI_ISL_18830726	5/2/2023	1/27/2024	Algiers
EPI_ISL_18830727	4/18/2023	1/27/2024	Algiers
EPI_ISL_18830728	4/25/2023	1/27/2024	Algiers
EPI_ISL_18830729	4/18/2023	1/27/2024	Algiers
EPI_ISL_18830730	4/3/2023	1/27/2024	Algiers
EPI_ISL_18830731	4/30/2023	1/27/2024	Algiers

EPI_ISL_18830732	5/6/2023	1/27/2024	Algiers
EPI_ISL_18830733	3/28/2023	1/27/2024	Oran
EPI_ISL_18830734	4/19/2023	1/27/2024	Oran
EPI_ISL_18830735	4/27/2023	1/27/2024	Tebessa
EPI_ISL_18830737	4/26/2023	1/27/2024	Tebessa
EPI_ISL_18830822	5/23/2023	1/27/2024	Tebessa
EPI_ISL_3161806	5/10/2021	8/1/2021	Ouargla
EPI_ISL_3161810	6/20/2021	8/1/2021	Algiers
EPI_ISL_3375624	6/14/2021	8/13/2021	Medea
EPI_ISL_3375625	6/14/2021	8/13/2021	Algiers
EPI_ISL_3375626	6/16/2021	8/13/2021	Bouira
EPI_ISL_3375627	6/22/2021	8/13/2021	Algiers
EPI_ISL_3375628	6/22/2021	8/13/2021	Algiers
EPI_ISL_3375629	6/24/2021	8/13/2021	Laghouat
EPI_ISL_3375630	6/24/2021	8/13/2021	Blida
EPI_ISL_3375631	6/27/2021	8/13/2021	Blida
EPI_ISL_3375632	6/30/2021	8/13/2021	Algiers
EPI_ISL_3375633	7/13/2021	8/13/2021	Algiers
EPI_ISL_3375634	7/13/2021	8/13/2021	Algiers
EPI_ISL_3718527	6/2/2021	8/26/2021	Algiers
EPI_ISL_3718528	6/2/2021	8/26/2021	Blida
EPI_ISL_3718531	4/12/2021	8/26/2021	Algiers
EPI_ISL_4004796	1/22/2021	9/7/2021	Sidi Bel Abbes
EPI_ISL_4004797	3/17/2021	9/7/2021	Sidi Bel Abbes
EPI_ISL_418241	3/2/2020	3/29/2020	Boufarik
EPI_ISL_418242	3/8/2020	3/29/2020	Blida
EPI_ISL_420037	3/2/2020	4/4/2020	Boufarik
EPI_ISL_5052203	7/22/2021	10/11/2021	Algiers
EPI_ISL_5052204	7/17/2021	10/11/2021	El Oued
EPI_ISL_5052205	7/8/2021	10/11/2021	Oran
EPI_ISL_5052211	7/19/2021	10/11/2021	Blida
EPI_ISL_7661152	12/10/2021	12/14/2021	Algiers
EPI_ISL_766861	6/29/2020	1/5/2021	Alger
EPI_ISL_766862	6/24/2020	1/5/2021	Bouira
EPI_ISL_766863	6/15/2020	1/5/2021	Tipaza
EPI_ISL_766864	7/15/2020	1/5/2021	Bouira
EPI_ISL_766865	6/19/2020	1/5/2021	S?©tif
EPI_ISL_766866	6/20/2020	1/5/2021	Bouira
EPI_ISL_766867	6/18/2020	1/5/2021	Alger
EPI_ISL_766868	6/1/2020	1/5/2021	Alger
EPI_ISL_766869	6/9/2020	1/5/2021	El Oued
EPI_ISL_766871	6/20/2020	1/5/2021	Laghouat
EPI_ISL_766872	6/19/2020	1/5/2021	Bouira
EPI_ISL_766873	6/22/2020	1/5/2021	Bordj-Bou-Arreridj

EPI_ISL_766874	6/21/2020	1/5/2021	Adrar
EPI_ISL_766875	6/17/2020	1/5/2021	Ouargla
EPI_ISL_8035611	11/20/2021	12/24/2021	Laghouat
EPI_ISL_8035612	11/20/2021	12/24/2021	Laghouat
EPI_ISL_8035613	11/20/2021	12/24/2021	Laghouat
EPI_ISL_8421063	9/17/2021	1/6/2022	Medea
EPI_ISL_8421065	9/10/2021	1/6/2022	Bejaia
EPI_ISL_8421066	9/12/2021	1/6/2022	Blida
EPI_ISL_8421067	9/12/2021	1/6/2022	Bouira
EPI_ISL_8421068	9/7/2021	1/6/2022	Blida
