# Supplementary material for: Tracing SARS-CoV-2 Evolution in Algeria: Insights from 2020 to 2023
Source: Viruses. 2026 Feb 18;18(2):258. doi: 10.3390/v18020258 (PMC12945117; doi:10.3390/v18020258)
Supplement: Supplementary file 1 [file viruses-18-00258-s001.zip › Supplementary material.pdf]

Supplementary material

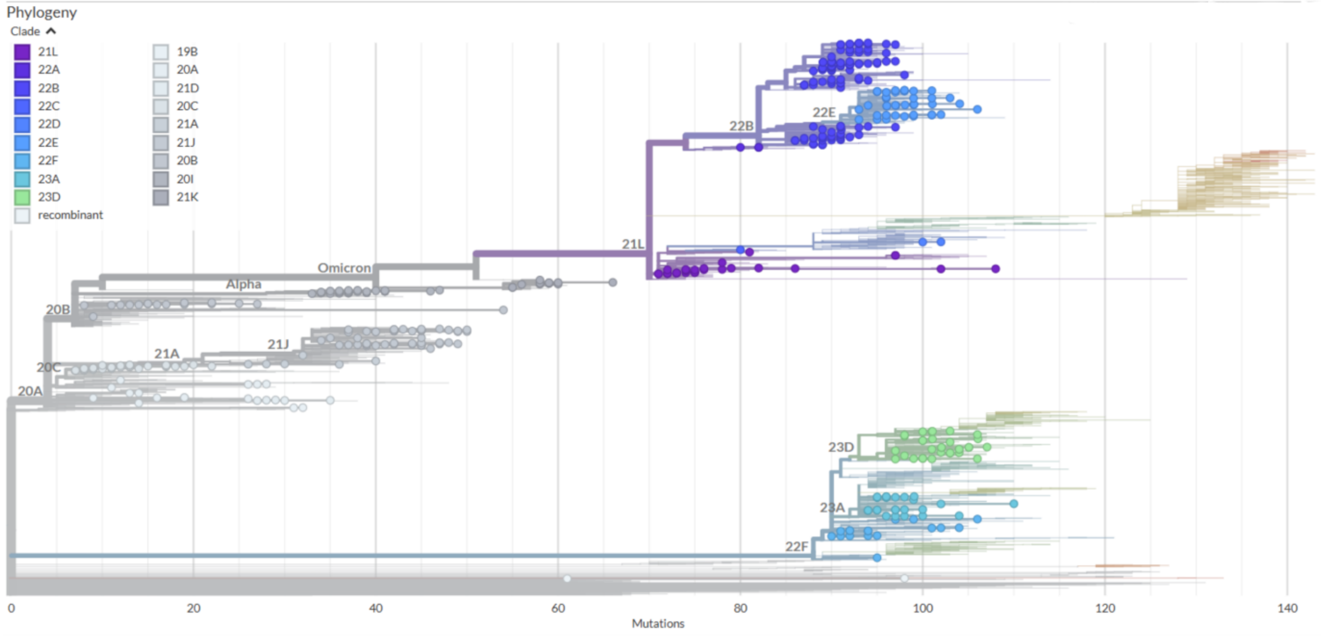

Supplementary Figure S1: Classification of Algerian sequences in the Nextclade phylo-genetic tree. The phylogeny was generated using Nextclade with GISAID as the database. A total of 449 Algerian sequences were introduced, and the colored dots indicate the clades to which each sequence belongs. The colors specified in the legend represent different clades, with grey indicating a recombinant sequence.

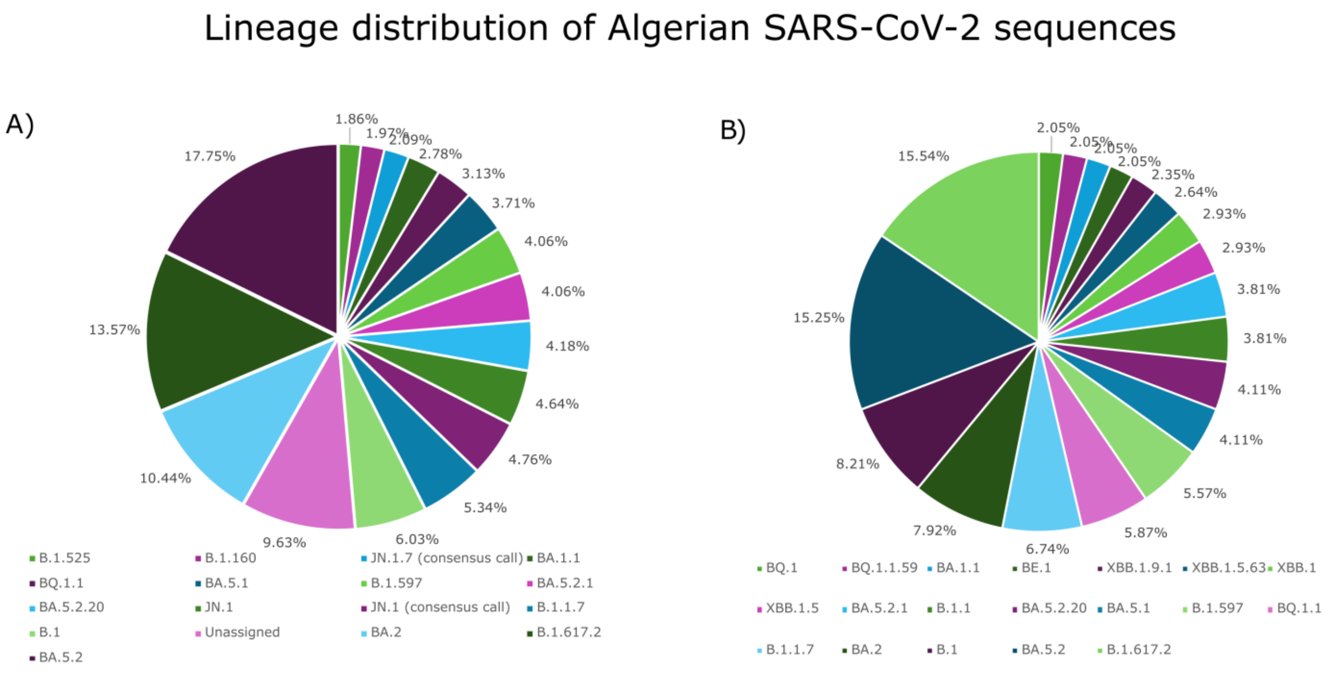

Supplementary Figure S2: Lineage distribution of Algerian SARS-CoV-2 sequences. (A) Lineage composition of all 1,218 Algerian SARS-CoV-2 sequences available in the GISAID database at the time of analysis. (B) Lineage composition of the curated dataset of 449 high-quality genomes. To enable meaningful comparisons between datasets of different sizes, lineage-frequency thresholds were scaled proportionally, and lineages representing fewer than approximately 1.2% of sequences were omitted. Each color represents a distinct lineage, or-dered in the legend by decreasing relative frequency.

ORF1a gene

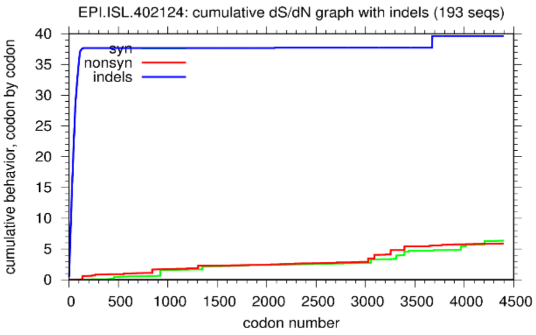

Spike gene

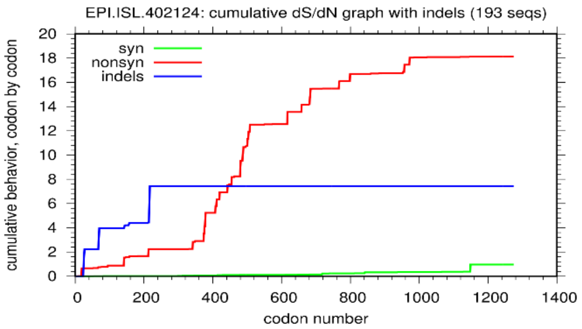

ORF1b gene

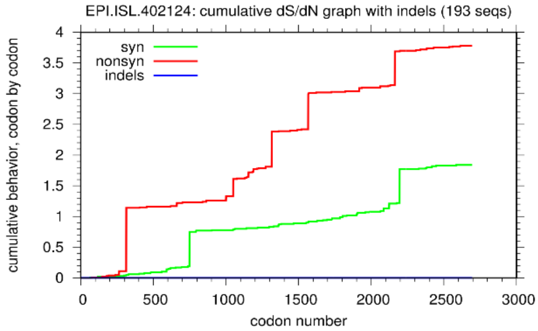

ORF3a gene

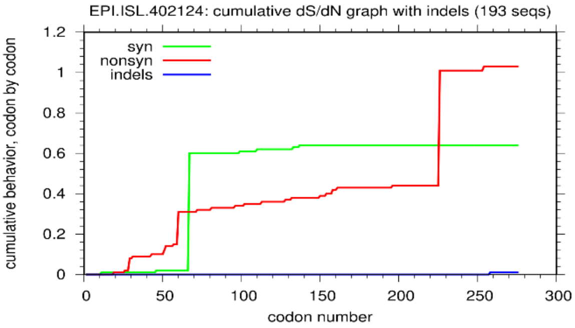

ORF7a gene

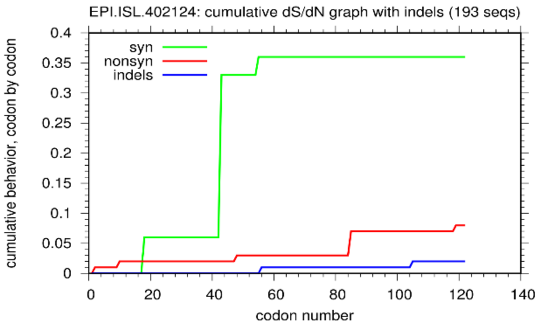

E gene

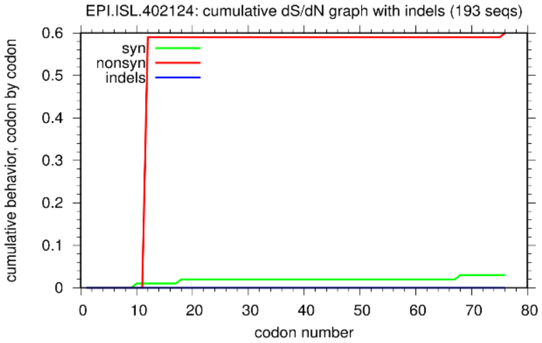

ORF7b gene

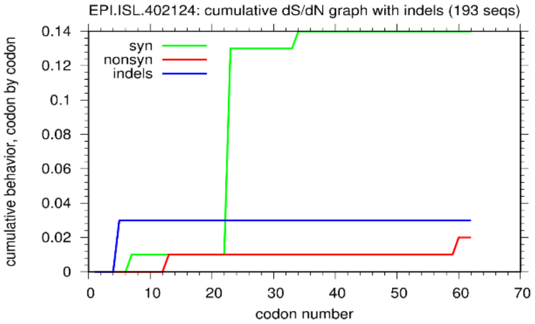

M gene

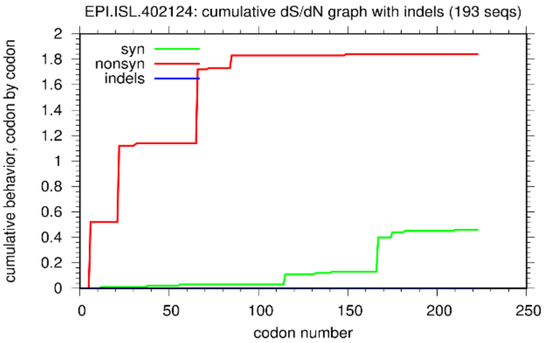

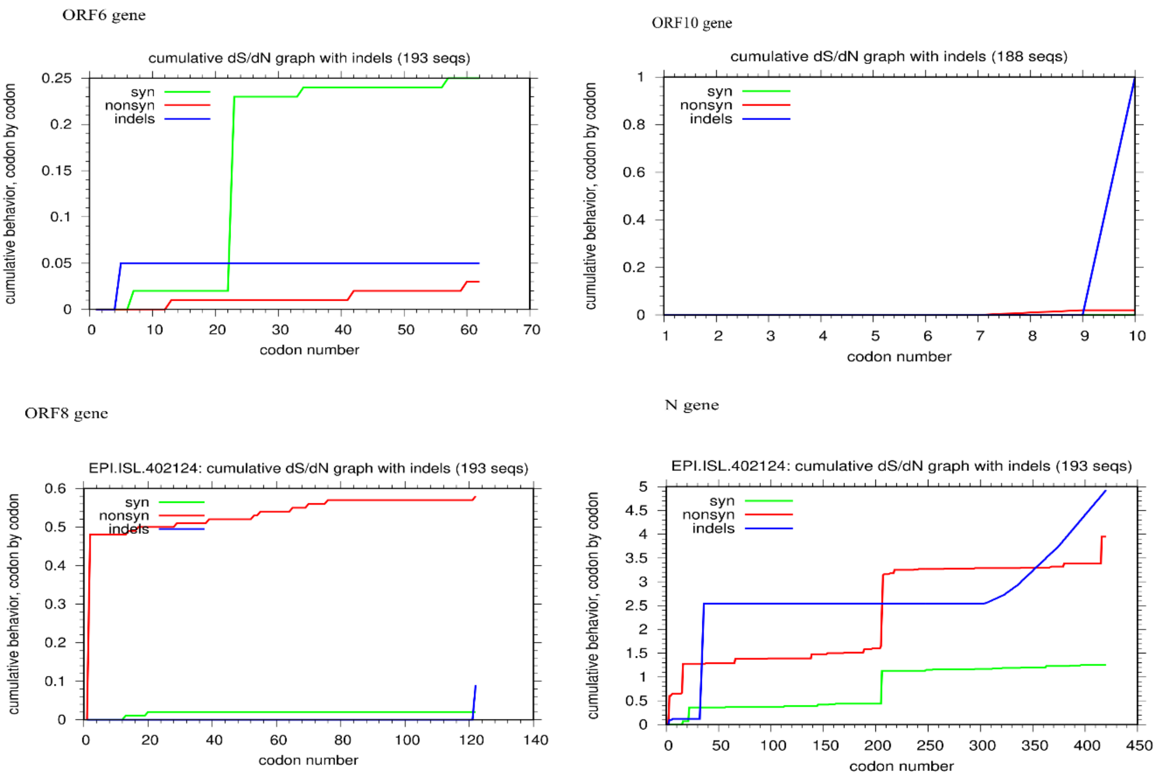

Supplementary Figure S3: Plots of insertion/deletion, synonymous, and nonsynonymous mutation rates for each nucleotide in the genes. The plot illustrates how genetic changes (substitutions) occur in two specific types, synonymous and nonsynonymous, across each analyzed gene. The accumulation of amino acid substitutions in 193 Algerian sequences was examined, including the SARS-CoV-2 reference sequence from Wuhan, China. In the graph, the X-axis represents the codons, and the Y-axis represents the number of substitutions. The red line corresponds to synonymous substitutions, the green line represents nonsynonymous substitutions, and the gray vertical line marks stop codons.

Supplementary Table S1: Mutations in SARS-CoV-2 proteins (complete set of Algerian sequences). Red indicates deleterious mutations, while green indicates neutral mutations.

Protein	Mutations	PredictSNP	MAPP	Phd-SNP	PolyPhen-1	PolyPhen-2	SIFT	SNAP
NSP1	S135R	**61%**	92%	66%	67%	43%	53%	62%
NSP2	Q376K	**60%**	82%	83%	67%	72%	46%	56%
	T183I	**74%**	72%	83%	67%	72%	45%	55%
NSP4	L264F	**83%**	65%	98%	67%	68%	77%	71%
	T327I	**83%**	85%	72%	67%	68%	77%	67%
	T492I	**83%**	76%	72%	67%	70%	71%	50%
NSP5	P132H	**75%**	77%	83%	67%	68%	67%	58%
NSP6	Q208R	**83%**	63%	58%	67%	68%	75%	67%
NSP12	T267M	**87%**	NA	59%	74%	81%	79%	85%
	N865H	**72%**	NA	58%	74%	60%	79%	62%
	Q866H	**75%**	NA	68%	67%	61%	53%	55%
	R233C	**72%**	48%	77%	74%	68%	79%	81%
NSP13	T127N	**83%**	79%	55%	67%	79%	67%	77%
	M233I	**83%**	70%	78%	67%	76%	68%	55%
	R392C	**63%**	70%	66%	67%	68%	53%	62%
NSP14	I42V	**83%**	70%	72%	67%	NA	66%	71%
NSP15	T112I	**83%**	89%	67%	73%	76%	NA	58%
SPIKE	A27S	**83%**	74%	83%	67%	64%	74%	77%
	V367F	**52%**	77%	68%	59%	56%	45%	56%
	G769V	**60%**	41%	55%	67%	59%	66%	56%
	Q677H	**63%**	NA	89%	59%	59%	71%	50%
	A27V	**65%**	71%	89%	67%	63%	46%	56%
	Q675H	**74%**	73%	89%	67%	74%	76%	56%
	Q784L	**74%**	72%	83%	67%	69%	77%	56%
	Y144D	**75%**	64%	66%	67%	68%	76%	89%

	V687I	**75%**	85%	72%	67%	40%	75%	61%
	Q134H	**75%**	75%	78%	67%	70%	61%	85%
	E484K	**83%**	75%	78%	67%	63%	77%	50%
	I844V	**83%**	74%	89%	67%	72%	90%	71%
	N679K	**83%**	NA	72%	67%	74%	78%	50%
	V213G	**63%**	64%	68%	67%	63%	79%	56%
	N658S	**83%**	78%	72%	67%	64%	89%	61%
	D796Y	**75%**	64%	68%	67%	69%	84%	58%
	Q954H	**74%**	75%	78%	67%	63%	65%	56%
NS3	S26L	**63%**	75%	83%	67%	73%	79%	50%
	Q57H	**76%**	77%	58%	74%	63%	46%	81%
	L129F	**72%**	76%	68%	59%	68%	45%	72%
	G100C	**51%**	66%	68%	67%	64%	71%	81%
	S60F	**60%**	63%	68%	67%	74%	43%	81%
	A23V	**74%**	46%	83%	67%	63%	71%	71%
	T151I	**87%**	92%	68%	59%	68%	79%	81%
	T223I	**87%**	92%	68%	59%	NA	79%	85%
E	T9I	**75%**	86%	51%	67%	71%	81%	61%
M	D3N	**75%**	79%	98%	67%	40%	70%	67%
	Q19E	**83%**	78%	83%	67%	72%	87%	67%
	L29F	**60%**	57%	68%	59%	61%	45%	55%
	A63T	**60%**	46%	68%	67%	55%	67%	55%
	I82T	**72%**	77%	58%	67%	63%	79%	81%
NS7b	T40I	**83%**	66%	NA	NA	NA	NA	NA
NS8	A51V	**65%**	63%	78%	67%	71%	43%	62%
	T26I	**60%**	62%	72%	67%	61%	43%	62%
	R52I	**72%**	84%	72%	74%	60%	79%	87%
N	A12G	**68%**	64%	72%	67%	50%	46%	77%
	R203K	**63%**	79%	78%	67%	60%	53%	72%
	R203M	**72%**	43%	78%	74%	81%	79%	85%

	G204R	**61%**	73%	83%	74%	81%	79%	72%
	G204P	**72%**	66%	72%	74%	81%	79%	56%
	S186Y	**72%**	82%	83%	74%	68%	79%	81%
	S194L	**65%**	77%	78%	59%	60%	79%	72%
	A251V	**61%**	88%	78%	59%	63%	79%	56%
	S202N	**55%**	56%	83%	59%	65%	65%	81%
	A398V	**60%**	92%	89%	67%	68%	53%	67%
	D63N	**60%**	63%	89%	67%	63%	53%	56%
	A211V	**74%**	64%	78%	67%	47%	76%	67%
	M210I	**83%**	64%	83%	67%	79%	76%	67%
